# On the Mortality at Different Ages in Certain Diseases

**Published:** 1844-10

**Authors:** Alexander Watt

**Affiliations:** City Statist


*On the mortality at different ages in certain diseases.*—By Alexander Watt, L.L.D., City Statist.*—In a paper which was published in the proceedings of the Glasgow Philosophical Society last year, I showed, from the returns obtained from different towns in England and Scotland, that the amount of deaths by various diseases is nearly identical at the same ages, and that whatever the total amount of deaths by each disease may be, the proportion which the deaths falling at certain pe-

* From the proceedings of the Philosophical Society of Glasgow, Jan. 3, 1844.

riods of life bears to the whole deaths by these respective diseases remains the same. The examples given in that paper related to fever, measles, small-pox, and bowel complaints. As the law which was deduced from these examples appeared to be of great interest, it became an important point to determine whether it was of more general application, since a knowledge of the specific laws of mortality by each disease at different ages, by determining more clearly the nature and operation of the disease, may be expected to lead both to improved modes of medical treatment, and to facilitate the introduction of such sanitary regulations as would insure to the inhabitants of our cities one of the most important of social blessings—a healthy population. Through the kindness of William Mills, Esq., formerly lord provost of Glasgow, I have been enabled to compare my former tables with a data which I have calculated from the mortality bills of New York and Philadelphia. From such comparative results we are enabled to distinguish, at a glance, the modifications which diseases undergo in the different climates. One of the most interesting diseases is typhus, on account of its frequent occurrence in this country, and more especially in Glasgow, where it seems to serve as a test of destitution.

*Fever.*—In the bills of mortality for New York and Philadelphia, the mortality by the different species of fever being judiciously given separately, we are enabled to select the species corresponding with those registered under the head of fever in the Scottish towns. The following table exhibits the comparative mortality from fever in Edinburgh and Glasgow, and from fevers, exclusively of puerperal and scarlet fevers, in New York and Philadelphia:

| | Edinburgh. | Glasgow. | N. York. | Philad. | Manch. |
|---|---|---|---|---|---|
| Deaths under 5 years to the whole deaths by fever | per cent. 12.41 | per cent. 12.07 | per cent. 15.67 | per cent. 17.34 | per cent. 16.08 |
| Deaths, under 20 years | 29.74 | 29.05 | 30.22 | 33.03 | 38.48 |
| Ditto, 20 and upwards | 70.25 | 70.94 | 69.77 | 66.96 | 61.51 |

In the above table the per centage for New York was deduced from 1,416 cases, and that of Philadelphia from 663 cases, The greatest difference appears to be at the lowest ages, where the mortality in America is higher. Similar results frequently occur in this country when the disease is less prevalent, as in 1842, in Glasgow, when the mortality under 5 years was 18.58. During that year it is well known that there was a smaller proportion of deaths by typhus than usual.

*Measles.*—The following table affords an extended illustration of the same law, which was pointed out in the last paper published in the "proceedings," and shows that the number who die of measles is nearly the same at the same ages in different towns:

| | Glasgow. | Edinburgh. | New York. | Philadelphia. |
|---|---|---|---|---|
| Under 2 years | 52.76 | 60.25 | 47.48 | 45.76 |
| " 5 " | 88.08 | 92.30 | 90.09 | 89.83 |
| " 20 " | 99.35 | 99.67 | 98.27 | 99.43 |
| Above 20 " | 0.64 | 0.32 | 1.72 | 0.56 |

The total amount of deaths in each of these towns was very different, and yet it will be observed that the proportions of deaths at the different ages to the whole deaths by measles are very nearly the same in each of these towns, the variation being chiefly at ages under two years.

---

*Scarlet Fever.*—The following table exhibits the per centage proportionate amount of deaths by scarlet fever, at different ages, in various towns, to the whole deaths by that disease in each town respectively:

| | Glasgow. | New York. | Philadelphia. |
|---|---|---|---|
| Under 2 years | 35.40 | 30.12 | 30.69 |
| " 5 " | 70.15 | 76.75 | 75.49 |
| " 20 " | 97.95 | 97.39 | 97.77 |
| Above 20 " | 2.04 | 2.60 | 2.22 |

Similar examples to the above are given for other towns of England and Scotland in the volume of the "British Association Transactions" for 1842, and from them it appears that the same law is applicable in all the localities examined. That the proportions of death by scarlet fever, at the different ages to the whole deaths, should be so nearly the same in New York and Philadelphia is remarkable, and although the amount for the lower ages differs from Glasgow, there is, no doubt, sufficient reason for this variation.

---

*Small-Pox.*—In this table we have compared the proportionate amount of deaths by small-pox, per cent., at different ages, in various towns, to the whole deaths by that disease in each town respectively:

| | Glasgow. | Edinburgh. | New York. | Philadelphia. |
|---|---|---|---|---|
| Under 2 years | 57.76 | 53.24 | 34.11 | 34.39 |
| " 5 " | 85.72 | 82.68 | 58.66 | 57.14 |
| " 20 " | 95.12 | 95.23 | 72.74 | 77.24 |
| Above 20 " | 4.87 | 4.76 | 27.25 | 22.75 |

From this table it appears that the proportion of deaths by small-pox to the whole amount of deaths by that disease in New York and Philadelphia, at the same ages, is very different from the proportion of deaths by the same disease in the towns of this country, the proportions under two years of age being above 23 per cent. less in New York and Philadelphia than in Glasgow. There is, of course, a corresponding increase in the proportion of deaths at the higher ages; yet it must be observed that the proportion of deaths by this disease at the early ages is the same in Philadelphia as it is at New York, affording another strong proof that there are physical laws which regulate the amount of deaths at different ages by the various diseases, when unimpeded by local causes. It is highly probable that inattention to early vaccination may be the immediate cause of a greater mortality at the higher ages in America than in this country. A difference in this respect exists also between the towns of England and Scotland. The proportion of deaths above twenty years of age by small-pox, in Manchester, amounts to 1.687 per cent. of the whole deaths by that disease, and to 2.316 per cent. in Liverpool; whereas the proportion above that age cut off by small-pox amounts to 4.479 per cent. of the whole deaths by that disease in Glasgow, and to 4.761 per cent. in Edinburgh. However much this effect in Glasgow and Edinburgh is produced by inattention to vaccination, the evil is very much the same in both cities, so far as the proportion at the higer ages is taken into account. It appears, also, from the returns, that the proportion of deaths by small-pox to the population in Edinburgh, is not half so great as that in Glasgow; and, as small-pox is much more destructive in some years than in others, and as the comparison only extends over three years for Edinburgh, and over five years for Glasgow, this comparison of the total amount of deaths by small-pox may be more favorable to Edinburgh than in ought to be.

---

*Hooping-Cough.*—This table shows the amount of deaths from hooping-cough, under and above certain ages, in different towns, and the proportion which the amount of deaths at these ages bears to the whole amount of deaths by that disease in each town respectively:

| | Glasgow. | Edinburgh. | New York. | Philadelphia. | Birmingham. |
|---|---|---|---|---|---|
| Under 2 years | 66.37 | 66.38 | 67.52 | 77.48 | — |
| " 5 " | 91.52 | 92.87 | 93.51 | 95.03 | 93.49 |
| " 20 " | 99.77 | 100.00 | 99.78 | 100.00 | 100.00 |
| Above 20 " | 0.22 | 0.00 | 0.21 | 0.00 | |

Some of these cases above twenty years should possibly not be classed with hooping-cough. One case, in Glasgow, is stated as being between 40 and 50, and another between 50 and 60. A case above twenty years, given in the New York tables, occurred in 1840, and is recorded as being between 30 and 40 years of age.

---

*Remarks on the Table.*—The data which have been adduced seem to demonstrate that the proportions in the amount of deaths under any given age, by the diseases which have been selected for consideration, viz., fevers, measles, scarlet fever, small-pox, and hooping-cough, to the whole amount of deaths by each disease respectively, are almost identical, although the same amount of deaths by the same disease is very different in each city. In some instances, where the circumstances of the people vary much from each other, a corresponding variation takes place in the mortality at the same ages. This is peculiarly exemplified by the deductions in reference to small-pox from the New York and Philadelphia mortality bills; but, notwithstanding the great difference between the results in America and in Scotland, in the mortality from this disease, it is important to observe that the proportion of the deaths by this disease in New York exactly corresponds with those for the same age in Philadelphia, the circumstances of these towns in relation to small-pox, being much alike. The variations in the higher ages may probably depend on causes capable of detection on further inquiry; and such interferences being allowed for the intimate correspondence of the results pointed out, cannot be looked upon as accidental, but as the result of precise laws, which regulate the amount of mortality at every age. Two causes must be especially considered as having a constant tendency to effect a certain variation in these results, viz., medical treatment, and a proper supply of nutritive food. Though it appears from these results that the medical practitioner does not possess that indiscriminate command over the life of his patient that has sometimes been ascribed to him, yet it is very apparent that, by judicious treatment, the medical man has much in his power in the way of placing the system of his patient in the most favorable circumstances for resisting the effects of the disease. If the patient, however, has been previously reduced by a scanty or improper diet, it may become difficult, perhaps even impossible, to supply the remedy under such circumstances, therefore it might be apprehended that even greater variations should occur in these proportions than are indicated by the details which have been discussed; but we must bear in mind that the practice of the medical man is not limited to individuals of a particular age, but is extended to whole families; and, in a similar manner, where destitution prevails, it very generally falls upon families at all ages, as well as upon particular persons.

An acquaintance with the laws of mortality which we have now considered, will not only aid us in arriving at a true knowledge of the sanitary conditions of towns, and enable us to point out the remedies for excessive mortality, but they will assist, also, in guiding the medical practitioner in the proper treatment of his patient. A knowledge of these laws is also necessary for the construction of proper annuity and life-insurance tables. Any calculations that are wholly founded on the average of life in other countries must necessarily be more or less falacious, as it is obvious that the average duration of human life must vary with the diseases which are most prevalent in the country; and it is well known that many countries, and even many districts, have diseases more or less peculiar to themselves, and differing in their law of motality.

*Lancet*, from *Bulletin Med. Science.*

